# Characterization and Modulation of Systemic Inflammatory Response to Exhaustive Exercise in Relation to Oxidative Stress

**DOI:** 10.3390/antiox9050401

**Published:** 2020-05-08

**Authors:** Katsuhiko Suzuki, Takaki Tominaga, Ruheea Taskin Ruhee, Sihui Ma

**Affiliations:** 1 Faculty of Sport Sciences, Waseda University, 2-579-15 Mikajima, Tokorozawa 359-1192, Japan; 2 Graduate School of Sport Sciences, Waseda University, Tokorozawa 359-1192, Japan; takaki.k-bbc@akane.waseda.jp (T.T.); ruhee@fuji.waseda.jp (R.T.R.)

**Keywords:** cytokine, neutrophil, macrophage, lipopolysaccharides (LPS), free fatty acids (FFA), Toll-like receptor (TLR), reactive oxygen species (ROS), anti-inflammatory effect of exercise, muscle and internal organ injury, immune suppression

## Abstract

Exhaustive exercise induces systemic inflammatory responses, which are associated with exercise-induced tissue/organ damage, but the sources and triggers are not fully understood. Herein, the basics of inflammatory mediator cytokines and research findings on the effects of exercise on systemic inflammation are introduced. Subsequently, the association between inflammatory responses and tissue damage is examined in exercised and overloaded skeletal muscle and other internal organs. Furthermore, an overview of the interactions between oxidative stress and inflammatory mediator cytokines is provided. Particularly, the transcriptional regulation of redox signaling and pro-inflammatory cytokines is described, as the activation of the master regulatory factor nuclear factor (erythroid-derived 2)-like 2 (Nrf2) is involved directly or indirectly in controlling pro-inflammatory genes and antioxidant enzymes expression, whilst nuclear factor-kappa B (NF-κB) regulates the pro-inflammatory gene expression. Additionally, preventive countermeasures against the pathogenesis along with the possibility of interventions such as direct and indirect antioxidants and anti-inflammatory agents are described. The aim of this review is to give an overview of studies on the systematic inflammatory responses to exercise, including our own group as well as others. Moreover, the challenges and future directions in understanding the role of exercise and functional foods in relation to inflammation and oxidative stress are discussed.

## 1. Introduction

Inflammation is usually accompanied by redness, fever, swelling, pain and loss of function, in which leukocyte infiltration and production of pro-inflammatory cytokines occur [1,2,3,4,5]. These inflammatory responses are considered as guards within our body to protect from external harmful stimuli such as microbial infection. However, prolonged exposure to inflammatory cytokines may induce chronic metabolic diseases like diabetes, cardiovascular diseases, chronic kidney diseases and cancer [3,5,6,7,8]. According to WHO, regular exercise is a planned, structured and purposeful movement of the body to maintain physical fitness and overall wellness [9]. Exercise has been treated as an anti-inflammatory therapy in recent times [6,7,8]. Therefore, regular exercise is considered as a kind of natural protector against chronic inflammatory diseases by releasing anti-inflammatory cytokines into the circulation [4,5,6]. Acute exercise may initiate a series of inflammatory cascades that depend on exercise intensity and duration. Intense exercise requires more energy than regular exercise. However, intense exercise causes a significant release of pro-inflammatory cytokines and free radicals from activated leukocytes, and leads to muscle damage and tissue injury [2,4,6,7,8,10]. Besides, during prolonged exercise, oxygen consumption increases and produces reactive oxygen species (ROS) via the electron transport chain (ETC) [7]. Numerous investigations have been carried out on the complex but intimate interactions among increased ROS and/or oxidative stress and inflammation. We have previously described the basic interaction and kinetics of cytokine production in response to exercise [4,11,12,13,14]. In this review, we will discuss the association between exercise-induced inflammatory response and tissue damage in different organs of the body. 

Key questions remain to be explored, such as what are the interactions of inflammatory mediator cytokines and oxidative stress? How are cytokines associated with the pathology of exercise-induced tissue damage? Which are useful biomarkers for assessing the effects of exercise? Here, besides introducing the basic background information on cytokines, we will discuss some research findings and the underlying mechanisms of cytokine release following exercise. Furthermore, overloaded skeletal muscle and other internal organs are examined to evaluate the association among cytokine responses, oxidative stress and tissue damage. Finally, we will discuss some prospective preventive countermeasures against the pathogenesis, and the possibility of anti-inflammatory interventions without harmful side-effects. The aim of this review is to give an overview of studies on the systematic inflammatory responses to exercise, including our own group as well as others.

## 2. Background Information of Cytokines

Designed to regulate inflammation and immune responses, cytokines are a diverse family of intercellular signaling molecules [4,11,13]. Cytokines are produced by numerous cells in tissues, where they function at very low concentration either in an autocrine or paracrine manner. Inflammation is a physiological protective response to initial tissue injury and occurs in damaged tissue which is also termed as localized inflammation. On the other hand, excessive localized inflammation releases cytokine into the circulation, which may become self-destructive, pathogenic, and sometimes fatal to the host [1,4,11,13]. The systemic inflammatory response syndrome (SIRS) is a clinical state, described by the systemic cytokine release (hypercytokinemia, known as cytokine storm). SIRS may introduce multiple organ damage in the pathology of severe invasion such as systemic infections, trauma, septic shock, ischemia-reperfusion injury, acute (adult) respiratory distress syndrome (ARDS), disseminated intravascular coagulation (DIC), and thermal injury [1,4,11,13]. The elevation of interleukin (IL)-6, IL-1β, IL-12, IL-17 and tumor necrosis factor (TNF)-α, have been observed in patients with sepsis; therefore, these pro-inflammatory mediators are considered to be the prime cytokines in the pathogenesis of systemic inflammation [1,5,13]. 

The three most influential cytokines operating in acute inflammation and/or SIRS are TNF-α, IL-1β and IL-6. In a typical inflammatory response model, TNF-α is the first cytokine, released systemically, and peaks within several hours after the beginning of inflammation, followed shortly thereafter IL-1β peaks, and then IL-6 appears [4,13,14]. These pro-inflammatory cytokines causes subsequent acute inflammatory responses, i.e., leukocytosis (neutrophilia) by inducing granulocyte colony-stimulating factor (G-CSF) and chemokines (abbreviated from chemotactic cytokines) such as IL-8 and monocyte chemotactic protein (MCP)-1 [1,4,5,11,13].

In the inflammatory conditions, anti-inflammatory responses are also induced to re-establish basal conditions. For example, to neutralize the pro-inflammatory cytokine cascade anti-inflammatory cytokines such as IL-10, IL-4 and IL-1 receptor antagonist (IL-1ra) are released into the circulation, which suppresses the pro-inflammatory cytokine production [5,6,14,15,16,17]. Cellular immunity is activated by IL-12, another major immunomodulatory cytokine [1,13,14,15]. IL-12 p70 is a bioactive heterodimer, consisting of two subunits: p35 and p40. In the absence of p35 expression, the IL-12 p40 homodimer and free p40 monomer acts as an IL-12 antagonist and does not mediate IL-12 activity but shares the activity with the other neutrophil activating cytokines such as IL-6 and IL-23 [1,17]. 

As seen above, there are some complexities in the cytokine network for regulating inflammation. However, it is worth mentioning that although blood samples have been used for measuring exercise-induced systemic cytokine secretion and other inflammatory mediators, analyses of urine samples may also be a feasible, reliable and a non-invasive way to observe cytokine dynamics [4,13,16,17,18,19,20].

## 3. Dynamics and Sources of Cytokines in Response to Exercise

Intensive endurance exercise perturbs the homeostasis of the immune system, which results in leukocytosis due to neutrophilia in the systemic circulation and immune suppression [2,12,18,19,20,21,22,23,24,25,26,27,28]. To identify the underlying mechanisms of these phenomena, considerable efforts have been focused on cytokine responses after exercise. Many studies have reported that IL-6, IL-10, IL-1ra and IL-8 increase after endurance exercise lasting longer than several hours, such as a marathon and triathlon [4,5,11,13,14,15,16,17,18,19,22,23,24,25,29,30,31]. However, during and after short-duration intensive exercise [4,13,26,27,28,32,33,34,35,36,37,38,39,40,41] and eccentric-contraction exercise [4,42,43,44,45,46,47], the response of these cytokines is negligible. These results suggest that these cytokine responses are related to exercise intensity and duration (physiological load/stress), rather than exercise-induced muscle damage [4,11,13,14,15,16,17,18,19,48]. 

IL-6 is a major cytokine which increases dramatically following endurance exercise. The representative regulators of IL-6 response to endurance exercise are low energy availability and heat stress which promote IL-6 response, and IL-6 response is subsequently correlated with stress hormone responses; however, they are suppressed by increased energy supply [4,5,15,29,30,49,50,51,52] and prior body-cooling interventions [4,35]. Furthermore, IL-6 enhances lipid availability such as free fatty acids (FFAs), which leads to endurance performance [4,5,15,27,53], whilst also promotes neutrophil migration and activation together with the release of anti-inflammatory cytokines such as IL-1ra and IL-10 [4,5,13,14,15,16,17,18,19]. Here, IL-10 is the most immunosuppressive cytokine, whilst IL-1ra (natural antagonistic cytokine) competes with IL-1 for receptor binding without inducing signal transduction. Endurance exercise also blocks cellular immune response, and causes susceptibility to infections by increasing plasma levels of IL-12 p40 and IL-4 [11,13,22,23].

**Figure 1:**
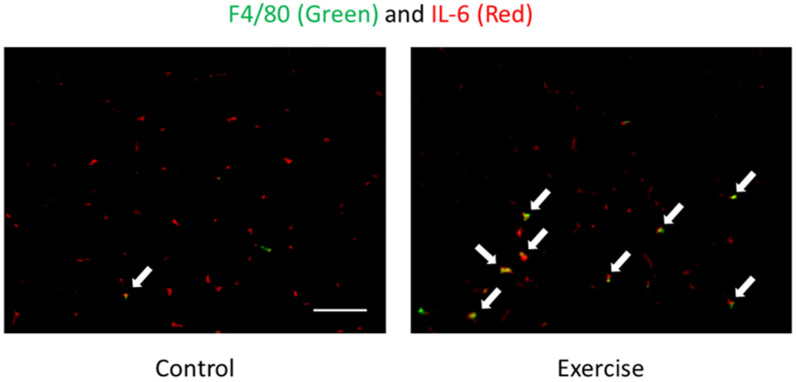
Localization of F4/80 (macrophages) (green) and IL-6 (red) of skeletal muscle after exercise detected by immunofluorescence staining [61]. Arrows (yellow) indicate F4/80 and IL-6 double positive cells. The signals of IL-6 were mainly observed in the interstitial space. Exercise increased F4/80 and IL-6 double positive cells but not IL-6 positive myocytes. This result suggests macrophages are one of the sources of IL-6.

Tissue infiltration of leukocytes is regulated by chemokines. IL-8 is an important neutrophil chemotactic and activation protein, and is released into the circulation under prolonged, intense exercise (e.g., marathon race) conditions [4,5,17,22,23,26,29,30,54], whereas short-duration intensive exercise (e.g., eccentric exercise or 10-min duration exhaustive exercise) also enhances plasma IL-8 concentration [4,13,26,47]. These findings suggest that both duration and intensity of exercise might be important for IL-8 release. IL-6 and G-CSF are also the regulator of neutrophil migration from the bone marrow to the circulation after exercise [13,26,31,37]. MCP-1 is one of the representative chemokine that facilitates the infiltration and activation of monocytes and macrophages. Many studies have reported upregulated MCP-1 concentration in both plasma and urine after a marathon race, and immediately after short-duration intensive exercise [13,17,18,20,31,54,55]. Although neutrophils are involved in exercise-induced muscle damage and inflammation, it has been recently notified that neutrophils recruited into skeletal muscle leads to exacerbating muscle injury, by up-regulating pro-inflammatory cytokines via the induction of macrophage infiltration with MCP-1 [56,57]. Whilst the specific organ origin of the circulatory release of MCP-1 is unknown, in the muscle tissue, neutrophils are one of the sources of MCP-1 following exercise [57]. Furthermore, in the muscle tissue, infiltrating neutrophils also produce IL-1β, and neutrophil-derived IL-1β has crucial roles in glucose uptake and endurance performance [58]. These findings suggest that infiltrating leukocytes may be one of the sources of cytokines and chemokines. Although endurance exercise induces various cytokines and chemokines, almost all of the original sources of increasing cytokines and chemokines are unknown. IL-6 is one of the cytokines released from skeletal muscle into circulation [5,59]. In the muscle tissue, myocytes are one of the sources of releasing IL-6 [60]. On the other hand, it has been demonstrated that infiltrating macrophages are another source of IL-6 following exercise (Figure 1) [56,61]. IL-6 secretion patterns are reported to be muscle fiber type-specific [60,62]; for example, the transcriptional level of IL-6 is much higher in the soleus (dominant slow-twitch fiber) than that in the gastrocnemius (dominant fast-twitch fiber) [61,63].

Similar to the above mechanisms, we have reported the adaptation phenomenon in which mobilization and activation of neutrophils were rare to occur alongside repeated exercise [21]. Moreover, muscle damage markers were inversely correlated with catecholamine secretion [26,64]. Indeed, stress further affects neutrophil ROS productivity [14,65]. In recent experiments using mice, neutrophil depletion using anti-neutrophil antibodies prevents infiltration of neutrophil in muscle tissue and muscle damage [57]. Moreover, macrophage depletion suppressed the infiltration of macrophages and production of pro-inflammatory cytokines in muscle tissue [56]. Our group has therefore been studying the anti-inflammatory effects of exercise and foods that suppress mobilization and activation of neutrophils and monocytes/macrophages as the sources of pro-inflammatory mediators and ROS [2,6,9,29,66,67,68,69,70,71,72,73,74,75,76,77,78,79,80].

On another front, it has been reported that the multifunctional IL-6 is a prime mediator of the anti-inflammatory effects of exercise [5]. In this manner, based on energy demand, contracting skeletal muscle produces IL-6 and releases it into the circulation, besides enhancing lipid availability in a similar manner to stress hormones [81]. Therefore, IL-6 was termed as a myokine and considered to support both energy supply and endurance performance [5]. However, although it was reported that high-intensity and/or endurance exercise induce IL-6, it was unclear whether IL-6 is released into the circulation in response to moderate exercise [25,82,83,84]. We have included a summary table of previous studies on exercise-induced cytokine dynamics including reports from our groups and others (Table 1). Then, we consider the mechanisms/triggers of exercise-induced inflammation. In fact, exercise-induced endotoxemia and organ damage are the main mechanisms/triggers of exercise-induced inflammation. We will discuss the mechanisms/triggers of exercise-induced inflammation in Section 4 and Section 5.

## 4. The Exercise-Induced Endotoxemia and Systemic Inflammation

Endotoxins, i.e., lipopolysaccharides (LPS), are constituents of enterobacteria and may cause endotoxemia or sepsis due to the nature of its translocation to the blood from gut [5,25,88]. It is also known as a systemic inflammation, and recently it was observed that a leaky gut is the onset of the pathogenic mechanisms of heat stroke [89,90]. Moreover, among the obese people, excessive adipose tissue releases FFAs with the breakdown of triglycerides [3,75]. Therefore, a cascade of inflammatory mediators is produced when LPS and FFAs bind to TLR4 of monocytes/macrophages. It can be presumed that infiltration of inflammatory cells into fat accumulated as a result of obesity becomes a site of prolonged production of inflammatory cytokines [6,91,92,93,94,95,96,97].

**Figure 2:**
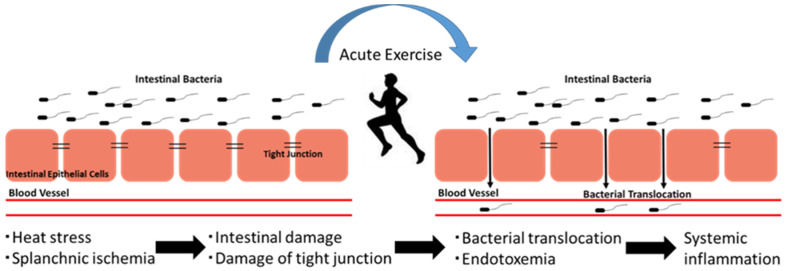
The concept of exercise-induced endotoxemia and systemic inflammation. Exercise induces intestinal barrier dysfunction and hyperpermeability. Subsequently, gut-derived bacteria translocate to the circulation and induce systemic inflammation.

The endotoxemia can be triggered either by pathogenic bacterial infection or by intestinal Gram-negative bacterial translocation. The hyperpermeability of the intestine can be increased with prolonged and strenuous exercises, which further increases the translocation of intestinal Gram-negative bacteria (Figure 2). It has been observed that the blood supply of muscle and cardiopulmonary system increases during prolonged exercise, while there is a reduction in the blood supply of the gastrointestinal system. Such a change in two systems due to the prolonged and strenuous exercise-induced redistribution of blood flow may cause gastrointestinal ischemia during exercise, whilst the reperfusion results in inflammation after exercise ceases [1,89,90,98,99,100]. The hyperpermeability of the intestines and gastrointestinal ischemia started due to exercise-induced redistribution of blood flow [89,90,100]. Besides that, the core body temperature may increase due to the prolonged and strenuous exercise that further leads to hyperthermia, as well as intestinal hyperpermeability [89,100]. In addition to that, during prolonged and strenuous exercise endotoxemia can happen due to the translocation of the intestinal Gram-negative bacteria into blood and increase circulatory bacterial LPS [1,89,90,100].

Under other conditions, exercise-induced endotoxemia produces a surge of pro-inflammatory mediators (prostaglandin (PG)E_2_, TNF-α, IL-1β and IL-6) by activated macrophages. These pro-inflammatory mediators/cytokines may cause fever that even result in exertional heat stroke. However, the pathogenic mechanism of heat stroke is associated with both hyperthermia and endotoxemia [1]. It is suggested that the pathogenic mechanism of heat stroke is covered by dual-pathway model of hyperthermia and endotoxemia [89].

Gut-derived endotoxin contributes to exercise-induced inflammatory response whereas it is unclear whether endotoxemia is a dominant cause of exercise-induced inflammatory response. In a review of Costa, et al., it is reviewed that moderate exercise durations (within 2 h duration) induces negligible rises in circulating endotoxin [98], whereas a large number of studies reported middle-duration exercise (within one hour of duration) induces systemic inflammation [2,4,13,29,100]. Furthermore, van Wijck, et al. have reported exercise-induced inflammatory responses (e.g., the increase of myeloperoxidase and calprotectin, which are neutrophil and macrophage activation markers), but not endotoxemia after one hour of cycling exercise [85]. Therefore, other factors may also contribute to the exercise-induced inflammatory response. Organ damage is a potential factor of systemic inflammatory response. In short-duration exercise (within one hour of duration), the induction of organ damage coincides with systemic inflammatory response [2,4,13,29,85]. Therefore, damaged organ-derived cytokines may accelerate systemic inflammatory responses. Further studies are necessary to elucidate the relationship between endotoxin and systemic inflammatory responses.

## 5. The Exercise-Induced Inflammation and Organ Damage

Intense exercise induces internal organ damage such as renal, hepatic and intestinal damage [16,17,18,19,20,24,85]. Exercise duration and intensity are important factors for the degree of organ damage. For example, long-duration exercise (e.g., marathon race) induces severe organ damage compared to middle-duration exercise (e.g., 1-h cycling) [16,17,18,19,20,24,85]. As described in Section 2, the degree of inflammatory response is dependent on exercise intensity and duration. These results suggest that the degree of organ damage is involved in the degree of inflammatory responses.

Because intense exercise increases blood supply of muscle, hypoperfusion of internal organs is induced, which results in exercise-induced organ damage. Organ damage induced by ischemia and trauma leads to systemic inflammatory responses, which result in multiple organ damage [4,11,13]. In damaged tissues, the expression of inflammatory cytokines such as IL-1β, IL-6 and TNF-α and leukocyte infiltration are observed [101,102,103]. Similarly, exercise increases the expression of cytokines such as IL-1β, IL-6 and TNF-α in kidney [104], liver [80] and intestine [105,106]. When organ damage is induced, damaged cells produce cytokines, chemokines and damage-associated molecular patterns (DAMPs), which promote leukocyte migration in damaged tissues. Then, infiltrating leukocytes accelerate further tissue damage [1,101,102,103]. Our previous studies show that depletion of neutrophils or macrophages inhibits exercise-induced muscle damage and the expression of IL-1β, IL-6 and TNF-α [56,57]. These studies suggest that infiltrating leukocytes may play an important role as a key regulator of organ damage.

During exercise, heat stress and dehydration augment hypoperfusion of internal organs [107]. These processes also augment exercise-induced inflammatory responses (e.g., the increase of IL-1β, IL-1ra, IL-6, IL-8, IL-10 and TNF-α) [4,35,36,98] and exercise-induced organ damage [108,109]. These results suggest that hypoperfusion of internal organs leads to organ damage and systemic inflammatory responses, which promote further organ damage.

## 6. Interaction of Cytokines and Oxidative Stress

The exercise-induced cytokine response is dramatic; for example, plasma IL-6 may increase over 100-fold after strenuous full-marathon races [4,5,13,15,18,22,23]. However, few indirect and direct evidence supports the interaction between oxidative stress and pro-inflammatory cytokine production. To identify the acute post-exercise effect, a time-course study employing incremental cycle exercise until volitional exhaustion was carried out among healthy sedentary adults, and pro-inflammatory cytokines and oxidative stress markers (TBARS) from plasma were measured. There it was reported that exercise-induced oxidative stress does not promote cytokine release over 30 min post-exercise [110]. Similarly, we conducted a repeated cycling exercise over 3 days (90 min/day), to analyze the adaptive mechanism regulating systemic inflammatory response of stressed body. The inflammatory response is the highest on day 1, within 30 min of a 90-min exercise where plasma IL-6 concentration increased significantly, whereas ROS production occurred after 60 min [26].

**Figure 3:**
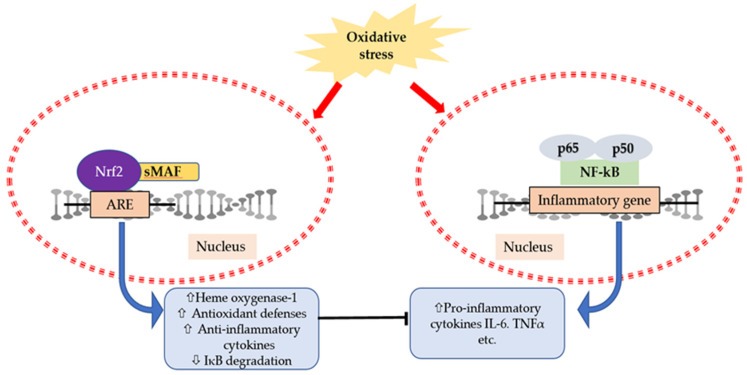
A schematic presentation of interactions between nuclear factor (erythroid-derived 2)-like 2 (Nrf2) and nuclear factor-kappa B (NF-κB) transcription factors. Under stressed conditions, Nrf2 translocates to the nucleus and activates the Nrf2/antioxidant responsive element (ARE) signaling pathway, which assists in the downstream regulation of NF-κB activity and expression of inflammatory genes.

Nuclear factor (erythroid-derived 2)-like 2 (Nrf2) and nuclear factor-kappa B (NF-κB) are two pivotal transcription factors in regulating cellular responses to inflammation and oxidative stress. In physiological conditions, Nrf2 remains suppressed in the cytoplasm by its repressor protein Keap1 [111,112]. Moreover, NF-κB also remains inactivated in the cytoplasm by the inhibitor of kappa B (IκB) kinase (IKK) complex [113]. Both Nrf2 and NF-κB are activated under stressed conditions and induce downstream stressors (Figure 3). Furthermore, Nrf2 dissociates from Keap1 and translocates to the nucleus followed by dimerizes with the small Maf family and binds to the ARE (antioxidant responsive element) in the promoter region. Therefore, there is an induction of Nrf2-related antioxidant genes such as heme oxygenase (HO)-1, and its transcriptional activation is actively involved in regulating inflammatory cytokines and chemokines [111,112]. Elevated levels of HO-1 can limit NF-κB activity by inhibiting IκBα degradation [114]. Previously it was reported that Nrf2 knockout mice showed an elevated activity of NF-κB and enhanced expression of inflammatory genes compared to wild-type mice [115,116]. In addition, the transcriptional activation of Nrf2 after a single bout of exhaustive exercise can reduce pro-inflammatory cytokine expression by inducing antioxidant enzymes [80]. Therefore, there is a crosstalk regarding the suppression of NF-κB signaling pathway and activation of Nrf2/ARE pathway [117].

The transcription factor NF-κB plays a central role during the activation of monocytes/macrophages, which are also responsible for cytokine secretion from contractile muscle [118]. The NF-κB signaling pathway is activated in a redox-sensitive manner after an acute bout of physical exercise, during muscular contraction, and decreases the inhibitory IκBα proteins [119]. Moreover, NF-κB binding was elevated for 2 h in the post-exercise period, before declining within 48 h [119]. Due to muscle contraction and relaxation we need a huge oxygen supply; therefore, superoxide, peroxide and hydroxyl radical production increases throughout the mitochondrial complexes. The pro-inflammatory cytokine production is triggered by mitochondrial ROS production, and they also act as signaling molecules for inflammation [120]. Though the cytokine response probably depends on the type, pattern and duration of exercise, increased lipid peroxidation (TBARS) levels have been observed immediately after a single short bout of maximum swimming [121]. All these data support that contractile activity is directly or indirectly responsible for NF-κB activation, either way causing inflammation.

## 7. Potential Strategy/Countermeasures to Reduce Exercise-Induced Inflammation and Oxidative Stress

**Figure 4:**
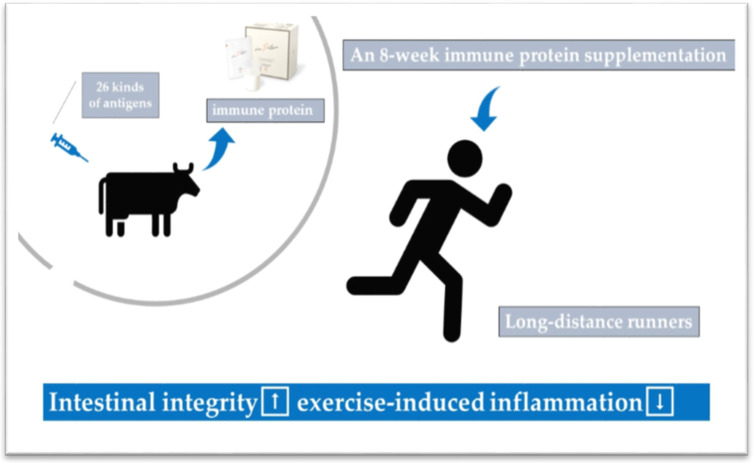
Protein from hyperimmunized milk showed protective effects towards exercise-induced inflammation [20].

Numerous preventive or therapeutic countermeasures have been identified against exercise-induced inflammation or oxidative stress. The systemic inflammatory response to exhaustive exercise can be attenuated by pre-exercise cooling [4,35,89], while post-exercise cooling may not have significant effects [4,45]. As shown in Figure 4, condensed protein from hyperimmunized milk that has shown promise toward maintaining gastrointestinal integrity showed anti-inflammatory effects after prolonged exercise [20]. Additionally, fluid intake is another effective strategy to prevent systemic induction of IL-6 and neutrophil activation markers [86,89], whilst intensive exercise in the menstruation phase of the menstruation cycle increases systemic inflammation [34]. During the daytime, morning exercise causes less release of IL-6 than evening exercise [27].

Moreover, some functional foods such as polyphenols or phytochemicals are attributed to enhance endurance capacity or attenuate inflammation. For example, supplementation of glucose may prevent infiltration of IL-6-producing macrophages in muscle tissues [61]. Carbohydrate ingestion also alters the physiological stress and inflammatory response among athletes [19]. On the other hand, a very low carbohydrate, high fat, ketogenic diet is reported to play roles in preventing exercise-induced organ and muscle damage [122,123]. Investigations of functional foods show that supplementation of polyphenols before uphill exhaustive running exercise may increase endurance capacity by upregulating blood glucose and muscle glycogen levels [79]. Besides, some functional foods can attenuate pro-inflammatory cytokine expression (IL-6, IL-1β and TNF-α), and lower the levels of two main inflammation regulatory enzymes (iNOS and COX2) [75,124]. Sulforaphane (SFN) is another emerging phytochemical that can significantly reduce exercise-induced oxidative stress [125,126] and inflammatory cytokines [76]. SFN is a natural antioxidant with many potential health benefits [127]. Dietary intake of SFN-rich food (broccoli sprout) has significant health benefits, and therefore protects our health from multiple metabolic disorders [128]. In addition to that, SFN treatment can improve muscle function by upregulating the expression of phase 2 enzymes (heme oxygenase 1) with Nrf2-dependent manner and reduced expression of inflammatory cytokines (TNF-α, IL-1β, and IL-6) by downregulating the expression of NF-κB and phosphorylated IκB in skeletal muscle [129]. Besides, SFN is a potential candidate to prevent inflammatory diseases by targeting monocytes/macrophages [130]. Apart from the supplementation, an 8-week low carbohydrate, high fat ketogenic diet (KD) can also have significant effects in reducing exercise-induced inflammation [62]. However, KD diet may have some deleterious consequences as it may cause glucose intolerance, whilst adverse effects of KD on the liver should be considered. We have also reported that an 8-week KD may increase intramuscular oxidative stress [123,124]. From a nutraceutical perspective, some of the probiotic supplementation may decrease clinical disorders and pro-inflammatory cytokine production [127,128,129,130], although some do not affect exercise-induced intestinal barrier dysfunction [99]. On the other hand, whilst carbohydrate supplementation enhances exercise-induced inflammation in the case of resistance/eccentric exercise [47], no adverse effects were observed in the case of endurance exercise [87]. A bunch of polyphenols which are derived from herbs, fruits and vegetables exhibit anti-inflammatory effects, and attenuate LPS-induced inflammation [1,2]. Particularly, curcumin ingestion after downhill running has been shown to reduced muscle inflammation in a mouse model of exercise-induced muscle damage [71]. Intestinal permeability increases following exercise, and endotoxemia occurs which induce systemic inflammation [88,89,98,99]. Therefore, other countermeasures such as ingesting some functional foods [1,12,66,131,132,133,134,135,136,137] for bioavailability, gut barrier protection and distribution, and appropriate immune responsiveness should be examined in future studies. Related reviews on neutrophil (leukocyte) responses to exercise are available elsewhere [2,12,32].

According to the above discussion, certain supplementations may alleviate exercise-induced oxidative stress such as green tea extract or polyphenols, and those antioxidants may increase exercise capacity and contribute to improving muscle health [69,72]. In any event, we can consider some combined and/or restricted applications. Whilst there is not enough current evidence to make specific recommendations, future studies in this area will contribute to the formation of such recommendations.

## 8. Conclusions

Neutrophils/macrophages and other pro-inflammatory cytokines/mediators can accumulate within the organs that are involved in tissue damage/dysfunction of not only skeletal muscle, but also liver, kidney and intestines. Such damage or organ dysfunction may refer to the pathogenesis of multiple organ failure in heat stroke and sepsis, and their underlying mechanisms. The harmful side effects on health can be reversible with proper countermeasures, for example, exercising in cool environments besides ingesting sufficient energy and fluids together with some functional food(s) which further help to maintain endurance performance. Moreover, a wide range of phytochemicals holding anti-inflammatory and antioxidant properties that suppress the NF-κB signaling pathway and activate the Nrf2/ARE pathway should be considered as countermeasures to exercise-induced inflammation. However, a future focus of research should consider how to apply these countermeasures appropriately. These countermeasures will help to reduce heat illness and also assure the introduction of new research findings for the prevention of stress and infection. The anti-inflammatory effects of exercise are well understood. In the near future, it is important to identify the effects of exercise from a pathological point of view, and to develop early prognostic markers [2,8,82,138,139,140,141,142].

## Figures and Tables

**Table 1:** The specific reports on the change of circulating cytokines, chemokines and immune cell activation markers following exercise in different research groups including ours.

References	Subjects	Exercise Protocols	Exercise Duration	Time Points	Measured Substances	The Changes of Substances
Suzuki, et al. [13]	10 male athletic students	Maximal exercise test by treadmill running	10.2 ± 1.7 min	IM, Post 1 h, Post 2 h	TNF-α, IL-1β, IL-2, IL-12, IFN-ɤ, IL-1ra, IL-4, IL-10, IL-6, G-CSF, GM-CSF, M-CSF, IL-8, MCP-1	IM: G-CSF, GM-CSF, M-CSF, MCP-1↑ Post 1 h: IL-1ra, IL-6, G-CSF, GM-CSF↑ Post 2 h: IL-1β, IL-4, G-CSF↑
Sugama, et al. [14,17]	14 male triathletes	Duathlon race (5-km running, 40-km cycling, 5-km running)	mean time; approx. 2 h	IM, Post 1.5 h, Post 3 h	TNF-α, IL-1β, IL-1ra, IL-2, IL-4, IL-6, IL-8, IL-10, IL-12, MCP-1, IL-17, IL-23, MPO, IL-12p40	IM: IL-1ra, IL-6, IL-8, IL-10, IL-12p40, MCP-1, MPO↑ IL-17, IL-23↓ Post 1.5 h: IL-1β, IL-1ra, IL-6, IL-8, MCP-1, MPO↑ Post 3 h: IL-1ra,↑
Suzuki, et al. [18]	10 male runners	Full marathon race	mean time; 2.62 h (rang, 2.55–68 h)	IM	TNF-α, IL-1β, IL-6, IL-8, IL-10, G-CSF, M-CSF, GM-CSF, MCP-1	IM: IL-6, IL-8, IL-10, G-CSF, M-CSF, MCP-1, MPO↑
Suzuki, et al. [19]	7 male triathletes	Duathlon race (5-km running, 40-km cycling, 5-km running)	mean time; approx. 2 h	IM	IL-6, IL-8, IL-10, IL-1ra, MCP-1	IM: IL-6, IL-8, IL-10, MCP-1↑
Suzuki, et al. [22]	16 male runners	Full marathon race	mean time; 2 h 34 min (rang, 2 h 25 min-2 h 40 min)	IM	IL-1β, IL-1ra, IL-2, IL-4, IL-6, IL-8, IL-10, IL-12, TNF-α, IFN-α, IFN-ɤ, G-CSF, GM-CSF, TGF-β1	IM: IL-1ra, IL-6, IL-8, IL-10, G-CSF↑ IL-4↓
Suzuki, et al. [24]	9 male triathletes	Ironman triathlon race (3.8-km swim, 180-km cycling, 42.2-km running)	mean time; 9 h 59 min	IM	IL-1ra, IL-6, IL-10, G-CSF, IL-12p40, IL-4, IL-1β	IM: IL-1ra, IL-6, IL-10, IL-12p40, G-CSF↑ Post 1 d: IL-1ra, IL-6, G-CSF↑
Suzuki, et al. [26]	8 male athletic students	Cycling with 90W power output	90 mim	Ex 30 min, 60 min, IM, Post 1 h, 3 h, 12 h	IL-1β, IL-6, IL-8, TNF-α, IFN-ɤ	IM: IL-6↑ Post 3 h: IL-6 Post 12 h: IL-6↑
Kim, et al. [27]	14 males with no regular exercise training	60% VO_2_max walking	60 min	IM, Post 2 h	IL-6, TNF-α, IL-1β	IM: IL-6↑
Nieman, et al. [29]	12 male and 4 female marathon runners	Treadmill running	3 h	IM	IL-6, IL-8, IL-10, IL-1ra	IM: IL-6, IL-8, IL-10, IL-1ra↑
Nieman, et al. [30]	15 trained male cyclists	75% VO_2_max cycling	2.5 h	IM, Post 12 h	IL-6, IL-8, IL-10, IL-1ra	IM: IL-6, IL-8, IL-10, IL-1ra↑
Nieman, et al. [31]	18 male and 3 female ultramarathon runners as the placebo group	160-km Western States Endurance Run	27.5 ± 0.6 h	IM	IL-6, IL-8, IL-10, IL-1ra, G-CSF, MCP-1, MIP-1β, TNF-α, MIF-1	IM: IL-6, IL-8, IL-10, IL-1ra, G-CSF, MCP-1, MIP-1β, TNF-α, MIF-1↑
Peake, et al. [33]	10 well-trained male runners	Running at 60% VO_2_max.	45 min	IM, Post 1 h, 24 h	IL-6, IL-8	IM: IL-6↑ Post 1 h:IL-6↑
Hayashida, et al. [34]	10 healthy sedentary females	Cycling at 75% of their individual anaerobic threshold	60 min	IM, Post 30 min	IL-6, Calprotectin, MPO	IM: IL-6↑ Post 30 min: Calprotectin, MPO↑
Peake, et al. [35]	10 well-trained male cyclists	Cycling at 60% VO_2_max + 16.1-km time trial	90 min	IM, R1: Post 35-40 min; R2: Post 80-85 min	Calprotectin, G-CSF, MPO, TNF-α, IL-1ra, IL-6, IL-8, IL-10	IM, R1 and R2: G-CSF, IL-8, Calprotectin, MPO, IL-10↑
Peake, et al. [36]	10 male cyclists	① 18.1 +/− 0.4 degrees C, 58% +/− 8% relative humidity, 90 min at approximately 60% VO_2_max and then completed a 16.1-km time trial②32.2 +/− 0.7 degrees C, 55% +/− 2% relative humidity, 90 min at approximately 60% VO_2_max and then completed a 16.1-km time trial	Cycling for 90 min and a time trial	IM	IL-6, IL-8, IL-10, G-CSF, Calprotectin, MPO	① and ②: IL-6, IL-8, IL-10, G-CSF, Calprotectin, MPO↑
Yamada, et al. [37]	12 male winter-sports athletes	A maximal exercise test on a treadmill (started at 220 m/min for the first 2 min and 220 m/min at a 4% grade for the next 2 min)	Mean running time: 10.3 ± 2.3 min	IM, Post 1 h, 2 h	G-CSF, IL-6	IM: G-CSF↑Post 1 h: IL-6↑
Mezil, et al. [39]	23 males	High intensity interval exercise	Total 6 min	Post 5 min, 1 h, 24 h	IL-1α, IL-1β, IL-6, TNF-α	Post 5 min: IL-1α, IL-1β, IL-6, TNF-α↑
Lira, et al. [40]	10 active males	① High intensity intermittent training ② Running at 70% maximal aerobic speed	Not described (total 5 km running)	IM, Post 1 h	IL-6, IL-10, TNF-α	Not changed
Brenner, et al. [41]	8 males	① All out cycling (equivalent to 90% VO_2_ max) ② Standard circuit-training routine ③ Cycling at 60–65% VO_2_ max	① 5 min ② Not described ③ 2 h	IM, Post 3 h, 24 h, 72 h	IL-6, TNF-α, IL-10	① Post 3 h: IL-10↓ Post 24 h: IL-10↓ Post 72 h: IL-10↓ ② Not changed ③ IM: IL-6↑, Post 3 h: IL-6, TNF-α↑ Post 24 h: TNF-α↑ Post 72 h: TNF-α↑
Kanda, et al. [42,43]	9 healthy males	10 sets of 40 repetitions of exercise at 0.5 Hz by the load corresponding to the half of body weight	Not described	Post 2 h, 4 h, 24 h, 48 h, 72 h, 96 h	TNF-α, IL-1β, IL-1ra, IL-2, IL-4, IL-6, IL-8,IL-10, IL-12, MCP-1, IL-17, IL-23,MPO, IL-12p40, IL-12 p70, IFN-γ, MCP-1, G-CSF, Calprotectin, C5a	Not changed
Scott, et al. [48]	10 active males	① Running at 55% VO_2_ max ② Running at 65% VO_2_ max ③ Running at 75% VO_2_ max	60 min	Ex 20 min, Ex 40 min, IM, Post 0.5 h, 1 h, 2 h, Post 3 h, 1 d, 2 d, 3 d	TNF-α, IL-6, IL-1ra	① Ex 40 min~Post 3 h: IL-6↑ ② Ex 40 min~Post 3 h: IL-6↑ Ex 40 min~Post: 1 h IL-1ra↑ ③ Ex 40 min~Post 3 h: IL-6↑ Ex 20 min~Post: 3 h IL-1ra↑
Nieman, et al. [54]	20 male cyclists	75 km cycling time trial	168 ± 26.0 min	IM	IL-6, IL-8, MCP-1	IM: IL-6, IL-8, MCP-1↑
van Wijck, et al. [85]	20 males	Cycling at 70% maximal workload	60 min	IM	MPO, Calprotectin	IM: MPO, Calprotectin↑
Suzuki, et al. [86]	6 well-trained male cyclists	Cycling at 60% VO_2_max	90 min	IM, Post 30 min	IL-1ra, MCP-1, IL-6, Calprotectin, MPO, IL-8, IL-10, IL-12p40	IM: IL-6↑; Post 30 min: Calprotectin↑
Tanisawa, et al. [87]	9 healthy males	Cycling at 70% VO_2_max	60 min	IM, Post 30 min, 1 h, 2 h	IL-6, G-CSF, MCP-1, IL-8, C5a, MPO, Calprotectin, Elastase	IM: IL-6, IL-8, MPO, Calprotectin, Elastase↑ Post 30 min: IL-6, G-CSF, MCP-1, IL-8, Calprotectin↑ Post 1 h: IL-6, G-CSF, Calprotecin↑ Post 2 h: IL-6, Calprotectin↑

VO_2_max, maximal oxygen uptake; IM, Immediately after exercise; Post X h, X indicates the lasting hour(s) from the completion of exercise; Ex, Time point during exercise; IL, Interleukin; TNF, Tumor necrosis factor; IFN, Interferon; IL-1ra, IL-1 receptor antagonist; G-CSF, Granulocyte colony-stimulating factor; GM-CSF, Granulocyte macrophage colony-stimulating factor; M-CSF, Macrophage colony-stimulating factor; MCP, Monocyte chemotactic protein; TGF, Transforming growth factor; MIP, Macrophage inflammatory protein; MIF, Macrophage migration inhibitory factor; MPO, Myeloperoxidase; C5a, Complement 5a; ↑, Increased after exercise; ↓, Decreased after exercise; ①, ②, ③, The result of each exercise protocol.

## References

[1] Hung Y.-L., Suzuki K. (2017). The pattern recognition receptors and lipopolysaccharides (LPS)-induced systemic inflammation. Int. J. Res. Stud. Med. Health Sci..

[2] Suzuki K. (2018). Involvement of neutrophils in exercise-induced muscle damage. Gen. Intern. Med. Clin. Innov..

[3] Ma S., Suzuki K. (2018). Toll-like receptor 4: Target of lipotoxicity and exercise-induced anti-inflammatory effect?. Ann. Nutr. Food Sci..

[4] Suzuki K. (2019). Characterization of exercise-induced cytokine release, the impacts on the body, the mechanisms and modulations. Int. J. Sports Exerc. Med..

[5] Pedersen B.K., Febbraio M.A. (2008). Muscle as an endocrine organ: Focus on muscle-derived interleukin-6. Physiol. Rev..

[6] Suzuki K. (2019). Chronic inflammation as an immunological abnormality and effectiveness of exercise. Biomolecules.

[7] Radak Z., Torma F., Berkes I., Goto S., Mimura T., Posa A., Balogh L., Boldogh I., Suzuki K., Higuchi M. (2019). Exercise effects on physiological function during aging. Free Radic. Biol. Med..

[8] Aw N.H., Canetti E., Suzuki K., Goh J. (2018). Monocyte subsets in atherosclerosis and modification with exercise in humans. Antioxidants.

[9] World Health Organization “Global strategy on diet, physical activity and health”. https://www.who.int/dietphysicalactivity/pa/en/.

[10] Cerqueira E., Marinho D.A., Neiva H.P., Lourenco O. (2020). Inflammatory effects of high and moderate intensity exercise—A systematic review. Front. Physiol..

[11] Suzuki K. (2018). Cytokine response to exercise and its modulation. Antioxidants.

[12] Peake J., Suzuki K. (2004). Neutrophil activation, antioxidant supplements and exercise-induced oxidative stress. Exerc. Immunol. Rev..

[13] Suzuki K., Nakaji S., Yamada M., Totsuka M., Sato K., Sugawara K. (2002). Systemic inflammatory response to exhaustive exercise: Cytokine kinetics. Exerc. Immunol. Rev..

[14] Sugama K., Suzuki K., Yoshitani K., Shiraishi K., Kometani T. (2012). IL-17, neutrophil activation and muscle damage following endurance exercise. Exerc. Immunol. Rev..

[15] Suzuki K., Nakaji S., Kurakake S., Totsuka M., Sato K., Kuriyama T., Fujimoto H., Shibusawa K., Machida K., Sugawara K. (2003). Exhaustive exercise and type-1/type-2 cytokine balance in special focus on interleukin-12 p40/p70. Exerc. Immunol. Rev..

[16] Sugama K., Suzuki K., Yoshitani K., Shiraishi K., Miura S., Yoshioka H., Mori Y., Kometani T. (2015). Changes of thioredoxin, oxidative stress markers, inflammation and muscle/renal damage following intensive endurance exercise. Exerc. Immunol. Rev..

[17] Sugama K., Suzuki K., Yoshitani K., Shiraishi K., Kometani T. (2013). Urinary excretion of cytokines versus their plasma levels after endurance exercise. Exerc. Immunol. Rev..

[18] Suzuki K., Nakaji S., Yamada M., Liu Q., Kurakake S., Okamura N., Kumae T., Umeda T., Sugawara K. (2003). Impact of a competitive marathon race on systemic cytokine and neutrophil responses. Med. Sci. Sports Exerc..

[19] Suzuki K., Shiraishi K., Yoshitani K., Sugama K., Kometani T. (2014). The effect of a sports drink based on highly branched cyclic dextrin on cytokine responses to exhaustive endurance exercise. J. Sports Med. Phys. Fit..

[20] Ma S., Tominaga T., Kanda K., Sugama K., Omae C., Hashimoto S., Aoyama K., Yoshikai Y., Suzuki K. (2020). Effects of an 8-week protein supplementation regimen with hyperimmunized cow milk on exercise-induced organ damage and inflammation in male runners: A randomized, placebo controlled, cross-over study. Biomedicines.

[21] Suzuki K., Naganuma S., Totsuka M., Suzuki K.J., Mochizuki M., Shiraishi M., Nakaji S., Sugawara K. (1996). Effects of exhaustive endurance exercise and its one-week daily repetition on neutrophil count and functional status in untrained men. Int. J. Sports Med..

[22] Suzuki K., Yamada M., Kurakake S., Okamura N., Yamaya K., Liu Q., Kudoh S., Kowatari K., Nakaji S., Sugawara K. (2000). Circulating cytokines and hormones with immunosuppressive but neutrophil-priming potentials rise after endurance exercise in humans. Eur. J. Appl. Physiol..

[23] Goh J.M., Lim C.L., Suzuki K., Schumann M., Ronnestad B. (2019). Effects of endurance-, strength-, and concurrent training on cytokines and inflammation. Concurrent Aerobic and Strength Training: Scientific Basics and Practical Applications.

[24] Suzuki K., Peake J., Nosaka K., Okutsu M., Abbiss C.R., Surriano R., Bishop D., Quod M.J., Lee H., Martin D.T. (2006). Changes in markers of muscle damage, inflammation and HSP70 after an Ironman triathlon race. Eur. J. Appl. Physiol..

[25] Peake J., Della Gatta P., Suzuki K., Nieman D. (2015). Cytokine expression and secretion by skeletal muscle cells: Regulatory mechanisms and exercise effects. Exerc. Immunol. Rev..

[26] Suzuki K., Totsuka M., Nakaji S., Yamada M., Kudoh S., Liu Q., Sugawara K., Yamaya K., Sato K. (1999). Endurance exercise causes interaction among stress hormones, cytokines, neutrophil dynamics, and muscle damage. J. Appl. Physiol..

[27] Kim H.K., Konishi M., Takahashi M., Tabata H., Endo N., Numao S., Lee S.K., Suzuki K., Kim Y.H., Sakamoto S. (2015). Effects of acute endurance exercise performed in the morning and evening on inflammatory cytokine and metabolic hormone responses. PLoS ONE.

[28] Suzuki K., Sato H., Kikuchi T., Abe T., Nakaji S., Sugawara K., Totsuka M., Sato K., Yamaya K. (1996). Capacity of circulating neutrophils to produce reactive oxygen species after exhaustive exercise. J. Appl. Physiol..

[29] Nieman D.C., Davis J.M., Henson D.A., Walberg-Rankin J., Shute M., Dumke C.L., Utter A.C., Vinci D.M., Carson J.A., Brown A. (2003). Carbohydrate ingestion influences skeletal muscle cytokine mRNA and plasma cytokine levels after a 3-h run. J. Appl. Physiol..

[30] Nieman D.C., Davis J.M., Henson D.A., Gross S.J., Dumke C.L., Utter A.C., Vinci D.M., Carson J.A., Brown A., McAnulty S.R. (2005). Muscle cytokine mRNA changes after 2.5 h of cycling: Influence of carbohydrate. Med. Sci. Sports Exerc..

[31] Nieman D.C., Henson D.A., Davis J.M., Dumke C.L., Gross S.J., Jenkins D.P., Murphy E.A., Carmichael M.D., Quindry J.C., McAnulty S.R. (2007). Quercetin ingestion does not alter cytokine changes in athletes competing in the Western States Endurance Run. J. Interf. Cytokine Res..

[32] Suzuki K. (2017). Exhaustive exercise-induced neutrophil-associated tissue damage and possibility of its prevention. J. Nanomed. Biother. Discov..

[33] Peake J.M., Suzuki K., Wilson G., Hordern M., Nosaka K., Mackinnon L., Coombs J. (2005). Exercise-induced muscle damage, plasma cytokines, and markers of neutrophil activation. Med. Sci. Sports Exerc..

[34] Hayashida H., Shimura M., Sugama K., Kanda K., Suzuki K. (2016). Exercise-induced inflammation during different phases of the menstrual cycle. Physiother. Rehabil..

[35] Peake J., Peiffer J.J., Abbiss C.R., Nosaka K., Okutsu M., Laursen P.B., Suzuki K. (2008). Body temperature and its effect on leukocyte mobilization, cytokines and markers of neutrophil activation during and after exercise. Eur. J. Appl. Physiol..

[36] Peake J., Peiffer J.J., Abbiss C.R., Nosaka K., Laursen P.B., Suzuki K. (2008). Carbohydrate gel ingestion and immunoendocrine responses to cycling in temperate and hot conditions. Int. J. Sport Nutr. Exerc. Metab..

[37] Yamada M., Suzuki K., Kudo S., Totsuka M., Nakaji S., Sugawara K. (2002). Raised plasma G-CSF and IL-6 after exercise may play a role in neutrophil mobilization into the circulation. J. Appl. Physiol..

[38] Peake J., Wilson G., Hordern M., Suzuki K., Yamaya K., Nosaka K., Mackinnon L., Coombes J.S. (2004). Changes in neutrophil surface receptor expression, degranulation, and respiratory burst activity after moderate- and high-intensity exercise. J. Appl. Physiol..

[39] Mezil Y.A., Allison D., Kish K., Ditor D., Ward W.E., Tsiani E., Klentrou P. (2015). Response of bone turnover markers and cytokines to high-intensity low-impact exercise. Med. Sci. Sports Exerc..

[40] Lira F.S., dos Santos T., Caldeira R.S., Inoue D.S., Panissa V.L.G., Cabral-Santos C., Campos E.Z., Rodrigues B., Monteiro P.A. (2017). Short-term high- and moderate-intensity training modifies inflammatory and metabolic factors in response to acute exercise. Front. Physiol..

[41] Brenner I.K., Natale V.M., Vasiliou P., Moldoveanu A.I., Shek P.N., Shephard R.J. (1999). Impact of three different types of exercise on components of the inflammatory response. Eur. J. Appl. Physiol. Occup. Physiol..

[42] Kanda K., Sugama K., Hayashida H., Sakuma J., Kawakami Y., Miura S., Yoshioka H., Mori Y., Suzuki K. (2013). Eccentric exercise-induced delayed-onset muscle soreness and changes in markers of muscle damage and inflammation. Exerc. Immunol. Rev..

[43] Kanda K., Sugama K., Sakuma J., Kawakami Y., Suzuki K. (2014). Evaluation of serum leaking enzymes and investigation into new biomarkers for exercise-induced muscle damage. Exerc. Immunol. Rev..

[44] Peake J.M., Roberts L.A., Figueiredo V.C., Egner I., Krog S., Aas S.N., Suzuki K., Markworth J.F., Coombes J.S., Cameron-Smith D. (2017). The effects of cold water immersion and active recovery on inflammation and cell stress responses in human skeletal muscle after resistance exercise. J. Physiol..

[45] Galvão D.A., Nosaka K., Taaffe D.R., Spry N., Kristjanson L.J., McGuigan M.R., Suzuki K., Yamaya K., Newton R.U. (2006). Resistance training and reduction of treatment side effects in prostate cancer patients. Med. Sci. Sports Exerc..

[46] Galvão D.A., Nosaka K., Taaffe D.R., Peake J., Spry N., Suzuki K., Yamaya K., McGuigan M.R., Kristjanson L.J., Newton R.U. (2008). Endocrine and immune responses to resistance training in prostate cancer patients. Prostate Cancer Prostatic Dis..

[47] Ross M.L., Halson S.L., Suzuki K., Garnham A., Hawley J.A., Cameron-Smith D., Peake J.M. (2010). Cytokine responses to carbohydrate ingestion during recovery from exercise-induced muscle injury. J Interf. Cytokine Res..

[48] Scott J.P.R., Sale C., Greeves J.P., Casey A., Dutton J., Fraser W.D. (2011). Effect of exercise intensity on the cytokine response to an acute bout of running. Med. Sci. Sports Exerc..

[49] Starkie R.L., Arkinstall M.J., Koukoulas I., Hawley J.A., Febbraio M.A. (2001). Carbohydrate ingestion attenuates the increase in plasma interleukin-6, but not skeletal muscle interleukin-6 mRNA, during exercise in humans. J. Physiol..

[50] Keller C., Keller P., Marshal S., Pedersen B.K. (2003). IL-6 gene expression in human adipose tissue in response to exercise--effect of carbohydrate ingestion. J. Physiol..

[51] Febbraio M.A., Steensberg A., Keller C., Starkie R.L., Nielsen H.B., Krustrup P., Ott P., Secher N.H., Pedersen B.K. (2003). Glucose ingestion attenuates interleukin-6 release from contracting skeletal muscle in humans. J. Physiol..

[52] Hashimoto H., Ishijima T., Hayashida H., Suzuki K., Higuchi M. (2014). Menstrual cycle phase and carbohydrate ingestion alter immune response following endurance exercise and high intensity time trial performance test under hot conditions. J. Int. Soc. Sports Nutr..

[53] Gudiksen A., Schwartz C.L., Bertholdt L., Joensen E., Knudsen J.G., Pilegaard H. (2016). Lack of skeletal muscle IL-6 affects pyruvate dehydrogenase activity at rest and during prolonged exercise. PLoS ONE.

[54] Nieman D.C., Zwetsloot K.A., Lomiwes D.D., Meaney M.P., Hurst R.D. (2016). Muscle glycogen depletion following 75-km of cycling is not linked to increased muscle IL-6, IL-8, and MCP-1 mRNA expression and protein content. Front. Physiol..

[55] Mansour S.G., Verma G., Pata R.W., Martin T.G., Perazella M.A., Parikh C.R. (2017). Kidney injury and repair biomarkers in marathon runners. Am. J. Dis..

[56] Kawanishi N., Mizokami T., Niihara H., Yada K., Suzuki K. (2016). Macrophage depletion by clodronate liposome attenuates muscle injury and inflammation following exhaustive exercise. Biochem. Biophys. Rep..

[57] Kawanishi N., Mizokami T., Niihara H., Yada K., Suzuki K. (2016). Neutrophil depletion attenuates muscle injury after exhaustive exercise. Med. Sci. Sports Exerc..

[58] Tsuchiya M., Sekiai S., Hatakeyama H., Koide M., Chaweewannakorn C., Yaoita F., Tan-No K., Sasaki K., Watanabe M., Sugawara S. (2018). Neutrophils provide a favorable IL-1-mediated immunometabolic niche that primes GLUT4 translocation and performance in skeletal muscles. Cell Rep..

[59] Steensberg A., Van Hall G., Osada T., Sacchetti M., Saltin B., Pedersen B.K. (2000). Production of interleukin-6 in contracting human skeletal muscles can account for the exercise-induced increase in plasma interleukin-6. J. Physiol..

[60] Hiscock N., Chan M.H.S., Bisucci T., Darby I.A., Febbraio M.A. (2004). Skeletal myocytes are a source of interleukin-6 mRNA expression and protein release during contraction: Evidence of fiber type specificity. FASEB J..

[61] Tominaga T., Ma S., Saitou K., Suzuki K. (2019). Glucose ingestion inhibits endurance exercise-induced IL-6 producing macrophage infiltration in mice muscle. Nutrients.

[62] Banzet S., Koulmann N., Simler N., Birot O., Sanchez H., Chapot R., Peinnequin A., Bigard X. (2005). Fibre-type specificity of interleukin-6 gene transcription during muscle contraction in rat: Association with calcineurin activity. J. Physiol..

[63] Ma S., Huang Q., Tominaga T., Liu C., Suzuki K. (2018). An 8-week ketogenic diet alternated interleukin-6, ketolytic and lipolytic gene expression, and enhanced exercise capacity in mice. Nutrients.

[64] Moreau D., Dubots P., Boggio V., Guilland J.C., Cometti G. (1995). Effects of electromyostimulation and strength training on muscle soreness, muscle damage and sympathetic activation. J. Sports Sci..

[65] Kuriyama T., Machida K., Suzuki K. (1996). Importance of correlations between phagocytic activity and superoxide production of neutrophils under conditions of voluntary exercise and stress. J. Clin. Lab. Anal..

[66] Peake J.M., Suzuki K., Coombes J.S. (2007). The influence of antioxidant supplementation on markers of inflammation and the relationship to oxidative stress after exercise. J. Nutr. Biochem..

[67] Suzuki K. (2018). Inflammatory response to exercise and its prevention. Curr. Top. Biochem. Res..

[68] Suzuki K., Ohno S., Suzuki Y., Ohno Y., Okuyama R., Aruga A., Yamamoto M., Ishihara K.O., Nozaki T., Miura S. (2012). Effect of green tea extract on reactive oxygen species produced by neutrophils from cancer patients. Anticancer Res..

[69] Ohno S., Ohno Y., Suzuki Y., Miura S., Yoshioka H., Mori Y., Suzuki K. (2015). Ingestion of *Tabebuia avellanedae* (Taheebo) inhibits production of reactive oxygen species from human peripheral blood neutrophils. Int. J. Food Sci. Nutr. Diet..

[70] Hung Y.L., Miyazaki H., Fang S.H., Li C., Suzuki K. (2018). The structural characteristics of green tea polyphenols on lipopolysaccharide-stimulated RAW cells. J. Nutr. Biol..

[71] Kawanishi N., Kato K., Takahashi M., Mizokami T., Otsuka Y., Imaizumi A., Shiva D., Yano H., Suzuki K. (2013). Curcumin attenuates oxidative stress following downhill running-induced muscle damage. Biochem. Biophys. Res. Commun..

[72] Takahashi M., Suzuki K., Kim H.K., Otsuka Y., Imaizumi A., Miyashita M., Sakamoto S. (2013). Effects of curcumin supplementation on exercise-induced oxidative stress in humans. Int. J. Sports Med..

[73] Li C.Y., Suzuki K., Hung Y.L., Yang M.S., Yu C.P., Lin S.P., Hou Y.C., Fang S.H. (2017). Aloe metabolites prevent LPS-induced sepsis and inflammatory response by inhibiting mitogen-activated protein kinase activation. Am. J. Chin. Med..

[74] Hung Y.L., Fang S.H., Wang S.C., Cheng W.C., Liu P.L., Su C.C., Chen C.S., Huang M.Y., Hua K.F., Shen K.H. (2017). Corylin protects LPS-induced sepsis and attenuates LPS-induced inflammatory response. Sci. Rep..

[75] Ma S., Yada K., Lee H., Fukuda Y., Iida A., Suzuki K. (2017). Taheebo polyphenols attenuate FFA-induced inflammation in murine and human macrophage cell lines as inhibitor of COX-2. Front. Nutr..

[76] Ruhee R.T., Ma S., Suzuki K. (2019). Sulforaphane protects cells against lipopolysaccharide-stimulated inflammation in murine macrophages. Antioxidants.

[77] Kawamura T., Suzuki K., Takahashi M., Tomari M., Hara R., Gando Y., Muraoka I. (2018). Involvement of neutrophil dynamics and function in exercise-induced muscle damage and delayed onset muscle soreness: Effect of hydrogen bath. Antioxidants.

[78] Hirata N., Ichimaru R., Tominari T., Matsumoto C., Watanabe K., Taniguchi K., Hirata M., Ma S., Suzuki K., Grundler F.M.W. (2019). Beta-cryptoxanthin inhibits lipopolysaccharide-induced osteoclast differentiation and bone resorption via the suppression of inhibitor of NF-κB kinase activity. Nutrients.

[79] Yada K., Suzuki K., Oginome N., Ma S., Fukuda Y., Iida A., Radak Z. (2018). Single dose administration of taheebo polyphenol enhances endurance capacity in mice. Sci. Rep..

[80] Ruhee R.T., Ma S., Suzuki K. (2020). Protective effects of sulforaphane on exercise-induced organ damage via inducing antioxidant defense responses. Antioxidants.

[81] Petersen A.M.W., Pedersen B.K. (2005). The Anti-inflammatory effect of exercise. J. Appl. Physiol..

[82] Takahashi M., Miyashita M., Kawanishi N., Park J.H., Hayashida H., Kim H.S., Nakamura Y., Sakamoto S., Suzuki K. (2013). Low-volume exercise training attenuates oxidative stress and neutrophils activation in older adults. Eur. J. Appl. Physiol..

[83] Ogawa K., Sanada K., Machida S., Okutsu M., Suzuki K. (2010). Resistance exercise training-induced muscle hypertrophy was associated with reduction of inflammatory markers in elderly women. Mediat. Inflamm..

[84] Hayashida H., Shimura M., Sugama K., Kanda K., Suzuki K. (2015). Effects of the menstrual cycle and acute aerobic exercise on cytokine levels. J. Sports Med. Doping Stud..

[85] van Wijck K., Lenaerts K., van Loon L.J., Peters W.H., Buurman W.A., Dejong C.H. (2011). Exercise-induced splanchnic hypoperfusion results in gut dysfunction in healthy men. PLoS ONE.

[86] Suzuki K., Hashimoto H., Oh T., Ishijima T., Mitsuda H., Peake J.M., Sakamoto S., Muraoka I., Higuchi M. (2013). The effects of sports drink osmolality on fluid intake and immunoendocrine responses to cycling in hot conditions. J. Nutr. Sci. Vitaminol..

[87] Tanisawa K., Suzuki K., Ma S., Kondo S., Okugawa S., Higuchi M. (2018). Effects of ingestion of different amounts of carbohydrate after endurance exercise on circulating cytokines and markers of neutrophil activation. Antioxidants.

[88] Lim C.L., Pyne D.B., Horn P., Kalz A., Saunders P., Peake J., Suzuki K., Wilson G., Mackinnon L.T. (2009). The effects of increased endurance training load on biomarkers of heat tolerance during intense exercise in the heat. Appl. Physiol. Nutr. Metab..

[89] Lim C.L., Suzuki K. (2017). Systemic inflammation mediates the effects of endotoxemia in the mechanisms of heat stroke. Biol. Med..

[90] Leon L.R., Helwig B.G. (2010). Heat stroke: Role of the systemic inflammatory response. J. Appl. Physiol..

[91] Kawanishi N., Mizokami T., Yada K., Suzuki K. (2018). Exercise training suppresses scavenger receptor CD36 expression in kupffer cells of nonalcoholic steatohepatitis model mice. Physiol. Rep..

[92] Kawanishi N., Niihara H., Mizokami T., Yada K., Suzuki K. (2015). Exercise training attenuates neutrophil infiltration and elastase expression in adipose tissue of high-fat-diet-induced obese mice. Physiol. Rep..

[93] Kawanishi N., Niihara H., Mizokami T., Yano H., Suzuki K. (2013). Exercise training attenuates adipose tissue fibrosis in diet-induced obese mice. Biochem. Biophys. Res. Commun..

[94] Kawanishi N., Mizokami T., Yano H., Suzuki K. (2013). Exercise attenuates M1 macrophages and CD8+ T cells in the adipose tissue of obese mice. Med. Sci. Sports Exerc..

[95] Kawanishi N., Yano H., Mizokami T., Takahashi M., Oyanagi E., Suzuki K. (2012). Exercise training attenuates hepatic inflammation, fibrosis and macrophage infiltration during diet induced-obesity in mice. Brain Behav. Immun..

[96] Kawanishi N., Kato Y., Yokozeki K., Sawada S., Sakurai R., Fujiwara Y., Shinkai S., Goda N., Suzuki K. (2018). Effects of aging on serum levels of lipid molecular species as determined by lipidomics analysis in Japanese men and women. Lipids Health Dis..

[97] Sell H., Habich C., Eckel J. (2012). Adaptive immunity in obesity and insulin resistance. Nat. Rev. Endocrinol..

[98] Costa R.J.S., Snipe R.M.J., Kitic C.M., Gibson P.R. (2017). Systematic review: Exercise-induced gastrointestinal syndrome—implications for health and intestinal disease. Aliment. Pharmacol. Ther..

[99] Pires W., Veneroso C.E., Wanner S.P., Pacheco D.A.S., Vaz G.C., Amorim F.T., Tonoli C., Soares D.D., Coimbra C.C. (2017). Association between exercise-induced hyperthermia and intestinal permeability: A systematic review. Sports Med..

[100] van Wijck K., Lenaerts K., Grootjans J., Wijnands K.A., Poeze M., van Loon L.J., Dejong C.H., Buurman W.A. (2012). Physiology and pathophysiology of splanchnic hypoperfusion and intestinal injury during exercise: Strategies for evaluation and prevention. Am. J. Physiol. Gastrointest. Liver Physiol..

[101] Jaeschke H. (2006). Mechanisms of liver injury. II. Mechanisms of neutrophil-induced liver cell injury during hepatic ischemia-reperfusion and other acute inflammatory conditions. Am. J. Physiol. Gastrointest. Liver Physiol..

[102] Sharfuddin A.A., Molitoris B.A. (2011). Pathophysiology of ischemic acute kidney injury. Nat. Rev. Nephrol..

[103] Luissint A.-C., Parkos C.A., Nusrat A. (2016). Inflammation and the intestinal barrier: Leukocyte-epithelial cell interactions, cell junction remodeling, and mucosal repair. Gastroenterology.

[104] Wu G.L., Chen Y.S., Huang X.D., Zhang L.X. (2012). Exhaustive swimming exercise related kidney injury in rats-protective effects of acetylbritannilactone. Int. J. Sports Med..

[105] Godínez-Victoria M., Drago-Serrano M.E., Reyna-Garfias H., Viloria M., Lara-Padilla E., Resendiz-Albor A.A., Sánchez-Torres L.E., Cruz-Hernández T.R., Campos-Rodriguez R. (2012). Effects on secretory IgA levels in small intestine of mice that underwent moderate exercise training followed by a bout of strenuous swimming exercise. Brain Behav. Immun..

[106] Hou P., Zhou X., Yu L., Yao Y., Zhang Y., Huang Y., Chen M., Yi L., Mi M. (2020). Exhaustive exercise induces gastrointestinal syndrome through reduced ILC3 and IL-22 in mouse model. Med. Sci. Sports Exerc..

[107] Laughlin M.H., Davis M.J., Secher N.H., van Lieshout J.J., Arce-Esquivel A.A., Simmons G.H., Bender S.B., Padilla J., Bache R.J., Merkus D. (2012). Peripheral circulation. Compr. Physiol..

[108] Snipe R.M.J., Khoo A., Kitic C.M., Gibson P.R., Costa R.J.S. (2018). The impact of exertional-heat stress on gastrointestinal integrity, gastrointestinal symptoms, systemic endotoxin and cytokine profile. Eur. J. Appl. Physiol..

[109] Sureda A., Mestre-Alfaro A., Banquells M., Riera J., Drobnic F., Camps J., Joven J., Tur J.A., Pons A. (2015). Exercise in a hot environment influences plasma anti-inflammatory and antioxidant status in well-trained athletes. J. Therm. Biol..

[110] Steinberg J.G., Ba A., Bregeon F., Delliaux S., Jammes Y. (2007). Cytokine and oxidative responses to maximal cycling exercise in sedentary subjects. Med. Sci. Sports Exerc..

[111] Ahmed S.M.U., Luo L., Namani A., Wang X.J., Tang X. (2017). Nrf2 signaling pathway: Pivotal roles in inflammation. Biochim. Biophys. Acta.

[112] Nemes R., Koltai E., Taylor A.W., Suzuki K., Gyori F., Radak Z. (2018). Reactive oxygen and nitrogen species regulate key metabolic, anabolic, and catabolic pathways in skeletal muscle. Antioxidants.

[113] Albensi B.C. (2019). What is nuclear factor kappa B (NF-κB) doing in and to the mitochondrion?. Front. Cell Dev. Biol..

[114] Wardyn J.D., Ponsford A.H., Sanderson C.M. (2015). Dissecting molecular cross-talk between Nrf2 and NF-κB response pathways. Biochem. Soc. Trans..

[115] Jin W., Wang H., Yan W., Xu L., Wang X., Zhao X., Yang X., Chen G., Ji Y. (2008). Disruption of Nrf2 enhances upregulation of nuclear factor-B activity, proinflammatory cytokines, and intercellular adhesion molecule-1 in the brain after traumatic brain injury. Mediat. Inflamm..

[116] Mao L., Wang H., Qiao L., Wang X. (2010). Disruption of Nrf2 enhances the upregulation of nuclear factor-kappaB activity, tumor necrosis factor, and matrix metalloproteinase-9 after spinal cord injury in mice. Mediat. Inflamm..

[117] Bellezza I., Mierla A.L., Minelli A. (2010). Nrf2 and NF-κB and their concerted modulation in cancer pathogenesis and progression. Cancers.

[118] Baeuerle P.A., Henkel T. (1994). Function and activation of NF-kappaB in the immune system. Ann. Rev Immunol..

[119] Ji L.L., Gomez-Cabrera M.C., Steinhafel N., Vina J. (2004). Acute exercise activates nuclear factor (NF)-κB signaling pathway in rat skeletal muscle. FASEB J..

[120] Bulua A.C., Simon A., Maddipati R., Pelletier M., Park H., Kim K.Y., Sack M.N., Kastner D.L., Siegel R.M. (2011). Mitochondrial reactive oxygen species promote production of proinflammatory cytokines and are elevated in TNFR1-associated periodic syndrome (TRAPS). J. Exp. Med..

[121] Cleto L.S., Oleto A., Sousa L., Barreto T.O., Cruz J.d.S., Penaforte C.L., Magalhães J.C.d., Franco J.d.S., Pinto K.M.d.C., Azevedo A.C.C. (2011). Plasma cytokine response, lipid peroxidation and NF-κB activation in skeletal muscle following maximum progressive swimming. Braz. J. Med. Biol. Res..

[122] Ma S., Suzuki K. (2018). Potential application of ketogenic diet to metabolic status and exercise performance: A review. EC Nutr..

[123] Ma S., Suzuki K. (2019). Keto-adaptation and endurance exercise capacity, fatigue recovery, and exercise-induced muscle and organ damage prevention. Sports.

[124] Ma S., Huang Q., Yada K., Liu C., Suzuki K. (2018). An 8-week ketogenic low carbohydrate, high fat diet enhanced exhaustive exercise capacity in mice. Nutrients.

[125] Oh S., Komine S., Warabi E., Akiyama K., Ishii A., Ishige K., Mizokami Y., Kuga K., Horie M., Miwa Y. (2017). Nuclear factor (erythroid derived 2)-like 2 activation increases exercise endurance capacity via redox modulation in skeletal muscles. Sci. Rep..

[126] Malaguti M., Angeloni C., Garatachea N., Baldini M., Leoncini E., Collado P.S., Teti G., Falconi M., Gonzalez-Gallego J., Hrelia S. (2009). Sulforaphane treatment protects skeletal muscle against damage induced by exhaustive exercise in rats. J. Appl. Physiol..

[127] de Figueiredo S.M., Binda N.S., Nogueira-Machado J.A., Vieira-Filho S.A., Caligiorne R.B. (2015). The antioxidant properties of organosulfur compounds (sulforaphane). Recent Pat. Endocr. Metab. Immune Drug Discov..

[128] Jeffery E.H., Araya M. (2009). Physiological effects of broccoli consumption. Phytochem. Rev..

[129] Sun C.-C., Li S.-J., Yang C.-L., Xue R.-L., Xi Y.-Y., Wang L., Zhao Q.-L., Li D.-J. (2015). Sulforaphane attenuates muscle inflammation in dystrophin-deficient Mdx mice via Nrf2-mediated inhibition of NF-κB signaling pathway. J. Biol. Chem..

[130] Pal S., Konkimalla V.B. (2016). Sulforaphane regulates phenotypic and functional switching of both induced and spontaneously differentiating human monocytes. Int. Immunopharmacol..

[131] Shing C.M., Peake J., Suzuki K., Okutsu M., Pereira R. (2007). Effects of bovine colostrum supplementation on immune variables in highly trained cyclists. J. Appl. Physiol..

[132] Shing C.M., Peake J.M., Suzuki K., Jenkins D.G., Coombes J.S. (2009). Bovine colostrum modulates cytokine production in human peripheral blood mononuclear cells stimulated with lipopolysaccharide and phytohemagglutinin. J. Interf. Cytokine Res..

[133] Abraham D., Feher J., Scuderi G.L., Szabo D., Dobolyi A., Cservenak M., Juhasz J., Ligeti B., Pongor S., Gomez-Cabrera M.C. (2019). Exercise and probiotics attenuate the development of Alzheimer’s disease in transgenic mice: Role of microbiome. Exp. Gerontol..

[134] Asadi A., Arazi H., Suzuki K. (2017). Effects of β-hydroxy-β-methylbutyrate-free acid supplementation on strength, power and hormonal adaptations following resistance training. Nutrients.

[135] Suzuki K., Takahashi M., Li C.Y., Lin S.P., Tomari M., Shing C.M., Fang S.H. (2015). The acute effects of green tea and carbohydrate co-ingestion on systemic inflammation and oxidative stress during sprint cycling. Appl. Physiol. Nutr. Metab..

[136] March D.S., Marchbank T., Playford R.J., Jones A.W., Thatcher R., Davison G. (2017). Intestinal fatty acid-binding protein and gut permeability responses to exercise. Eur. J. Appl. Physiol..

[137] Ogden H.B., Child R.B., Fallowfield J.L., Delves S.K., Westwood C.S., Layden J.D. (2020). The gastrointestinal exertional heat stroke paradigm: Pathophysiology, assessment, severity, aetiology and nutritional countermeasures. Nutrients.

[138] Shing C.M., Ogawa K., Zhang X., Nagatomi R., Peake J.M., Suzuki K., Jenkins D.G., Coombes J.S. (2007). Reduction in resting plasma granulysin as a marker of increased training load. Exerc. Immunol. Rev..

[139] Tsukamoto K., Suzuki K., Machida K., Saiki C., Murayama R., Sugita M. (2002). Relationships between lifestyle factors and neutrophil functions in the elderly. J. Clin. Lab. Anal..

[140] Ogawa K., Suzuki K., Okutsu M., Yamazaki K., Shinkai S. (2008). The association of elevated reactive oxygen species levels from neutrophils with low-grade inflammation in the elderly. Immun. Ageing.

[141] Roberts L., Suzuki K. (2019). Exercise and Inflammation. Antioxidants.

[142] Special Issue “Anti-Inflammatory and Antioxidant Effects of Dietary Supplementation and Lifestyle Factors”. https://www.mdpi.com/journal/antioxidants/special_issues/anti-inflammatory_antioxidant_effects.

